# Effects of hydroxytyrosol dose on the redox status of exercised rats: the role of hydroxytyrosol in exercise performance

**DOI:** 10.1186/s12970-018-0221-3

**Published:** 2018-04-27

**Authors:** Saad Al Fazazi, Rafael A. Casuso, Jerónimo Aragón-Vela, Cristina Casals, Jesús R. Huertas

**Affiliations:** “José Mataix” Institute of Nutrition and Food Technology, Biomedical Research Centre, Department of Physiology, University of Granada, laboratory 116. Av. del Conocimiento s/n, Armilla, 18100 Granada, Spain

**Keywords:** Polyphenols, ROS, Exercise, Hemoglobin, Oxidative stress, Antioxidants

## Abstract

**Background:**

Hydroxytyrosol (HT) is a polyphenol found in olive oil that is known for its antioxidant effects. Here, we aimed to describe the effects of a low and high HT dose on the physical running capacity and redox state in both sedentary and exercised rats.

**Methods:**

Male Wistar rats were allocated into 6 groups: sedentary (SED; *n* = 10); SED consuming 20 mg/kg/d HT (SED20; *n* = 7); SED consuming 300 mg/kg/d HT (SED300; n = 7); exercised (EXE; n = 10); EXE consuming 20 mg/kg/d HT (EXE20; n = 10) and EXE consuming 300 mg/kg/d HT (EXE300; n = 10). All the interventions lasted 10 weeks; the maximal running velocity was assessed throughout the study, whereas daily physical work was monitored during each training session. At the end of the study, the rats were sacrificed by bleeding. Hemoglobin (HGB) and hematocrit (HCT) were measured in the terminal blood sample. Moreover, plasma hydroperoxide (HPx) concentrations were quantified as markers of lipid peroxidation.

**Results:**

In sedentary rats, HT induced an antioxidant effect in a dose-dependent manner without implications on running performance. However, if combined with exercise, the 300 mg/kg/d HT dosage exhibited a pro-oxidant effect in the EXE300 group compared with the EXE and EXE20 groups. The EXE20 rats showed a reduction in daily physical work and a lower maximal velocity than the EXE and EXE300 rats. The higher physical capacity exhibited by the EXE300 group was achieved despite the EXE300 rats expressing lower HGB levels and a lower HCT than the EXE20 rats.

**Conclusions:**

Our results suggest that a high HT dose induces a systemic pro-oxidant effect and may prevent the loss of performance that was observed with the low HT dose.

## Background

Hydroxytyrosol (HT), the main polyphenol found in extra virgin olive oil, is in part responsible for the health-related effects of the Mediterranean Diet [1]. Polyphenols function as antioxidants due to their chemical structure composed of several hydroxyl groups on aromatic rings [2]. Accordingly, among other biological properties, HT has powerful in vivo antioxidant effects [3].

Moreover, recent data suggests that HT can improve mitochondrial function [4]. This feature has been previously described for different polyphenols [5–7]. However, despite mitochondria playing a key role in energy production during exercise [8], no evidence has been found to support any ergogenic potential of polyphenols [9, 10]. On the other hand, polyphenol supplementation has been reported to hinder exercise-induced skeletal muscle adaptations in both rodents and humans [11–13].

A plausible explanation comes from the antioxidant effects of polyphenols. Indeed, exercise adaptation is preceded by transient bursts of reactive oxygen species (ROS) production within contracting muscles [14]. These acute alterations in redox homeostasis lead to a chronic, systemic enhancement of antioxidant machinery by improving the function and content of both enzymatic and non-enzymatic antioxidants [14–16]. In addition, ROS are important molecular messengers that regulate redox-sensitive proteins involved in vascularization, mitochondrial biogenesis, immune response and growth factor signaling [17, 18]. Accordingly, subjects who show high oxidative stress in response to acute exercise are known to have greater training adaptations than those subjects who show low oxidative stress in response to the same exercise [19]. Therefore, blunting the acute ROS production within each training session with antioxidants may prevent some long-term exercise-induced adaptations. Accordingly, rats that were supplemented with 25 mg/kg/d HT and exercised for 10 weeks do not increase their performance when compared with sedentary rats [20]. This effect can be attributed to the antioxidant effects of HT, as the antioxidant effect of HT rises with the dosage from 10 mg/kg/d to 50 mg/kg/d HT in rodents [21].

However, under special conditions, polyphenols might become pro-oxidants [22]. In vitro studies show that as a result of their antioxidant activity, some oxidized metabolites are produced [22–24]. These metabolites induce oxidative damage to key proteins such as glutathione [23]. This paradoxical effect suggests that high polyphenol doses may induce a pro-oxidant environment. Similarly, high HT doses in vitro have been shown to increase ROS generation within tumor cells, leading to their apoptosis [25–27].

Since HT dosages up to 300 mg/kg/d are known to be safe for rodents [28], and a dosage of 20 mg/kg/d is known to exert an antioxidant effect [20, 21], we hypothesized that the consumption of a low (20 mg/kg/d) dosage of HT during endurance exercise would induce an antioxidant effect, while a high (300 mg/kg/d) dosage would reverse this effect by inducing systemic oxidative stress. Our purpose was to describe the effects of a low and high HT dose for 10 weeks on the physical capacity and redox state of both sedentary and exercised rats.

## Methods

### Animals

Male Wistar rats were purchased from Charles River (USA) at six weeks-old. The rats initially weighed 212 ± 13.5 g and were maintained in a well-ventilated room. This room was maintained under standard conditions of temperature (21 ± 2 °C) and relative humidity (40% to 60%) and under a reverse 12-h light/12-h dark cycle. Throughout the experimental period, all rats consumed water and standard chow ad libitum (2.9 kcal/g). Daily food and water intakes were monitored. All interventions lasted for 10 weeks. Rats were weighed weekly. Seventy-two hours after the last exercise was performed, rats were fasted overnight, anesthetized with pentobarbital and sacrificed by bleeding. The experiments were approved by the ethics committee of the University of Granada (Granada, Spain; n°: 28/06/2016/116).

### Hydroxytyrosol treatment

Rats receiving HT (Biomaslinic, S.L., Granada, Spain) were supplemented with a low dosage (20 mg/kg/d) or a high dosage (300 mg/kg/d) of HT. Supplementation began once the rats were allocated into the experimental groups and stopped 12 h before rats were euthanized. HT was diluted in water in an opaque drinking bottle. Water and HT were replaced every day in order to prevent HT oxidation. The dilution was adjusted weekly according to the weight of each rat and its average water intake. Based on our preliminary studies, this procedure is reliable for HT supplementation.

### Sedentary rats

Twenty-four rats were allocated into 3 groups: sedentary (SED; *n* = 10), SED consuming 20 mg/kg/d HT (SED20; *n* = 7), and SED consuming 300 mg/kg/d HT (SED300; n = 7). All the rats performed a maximal velocity performance test prior to the beginning of the study (Test 1) and after 10 weeks of treatment (Test 2). The maximal velocity test was a progressive intensity running test (PanLab treadmill for five rats, model LE 8710R) starting at a velocity of 22 cm/s and increasing by 5 cm/s every minute, similar to the previous study [29].

### Exercised rats

Thirty rats were allocated into 3 groups: exercised (EXE; *n* = 10), EXE consuming 20 mg/kg/d HT (EXE20; n = 10), and EXE consuming 300 mg/kg/d HT (EXE300; n = 10). Exercise training was divided into 2 mesocycles of 5 weeks each. Rats ran at 75% of their maximal velocity. Therefore, all rats allocated into exercised groups performed three maximal velocity tests (MVTs), as described above: Test 1 to establish their velocity during the first mesocycle; Test 2 to adjust the velocity for the second mesocycle; and Test 3 was performed 72 h after the last training session to evaluate maximal velocity capacity. After Test 1, rats were grouped by similar maximal velocity capacity as follows: EXE, 76.5 ± 10.12 cm/s; EXE20, 77.0 ± 8.50 cm/s; and EXE300, 77.4 ± 6.50 cm/s. Throughout the entire protocol, fatigue was defined as the point at which rats remained at the back of the treadmill on an electric shock pad for 5 s.

Each running mesocycle was identically designed. Rats started running for 20 min/d; this period was increased by 5 min every other day up to 65 min/d and was then maintained at 65 min/d until the end of the mesocycle. Moreover, physical work performed during each session was monitored as a reliable and continuous marker of exercise capacity [30]. We calculated the physical work performed by applying the formula: work (J) = force × vertical distance, where force = body weight (kg) × 9.8 m/s^2^, and vertical distance = speed (m/min) × time (min). Therefore, rats becoming fatigued before finishing the target time of a certain training session were removed from the treadmill and the time was recorded. This exercise protocol is known to induce both cellular and systemic redox adaptations [29].

### Feed efficiency

Feed efficiency measures the ability of an animal to transform the calories ingested into body weight. We followed the formula: feed efficiency = weight gain (g) × caloric intake (kcal)^− 1^ [31]. The caloric intake was calculated by the daily average food consumed in each cage containing five rats.

### Blood measurements

A portion of the blood obtained during the bleeding procedure was collected into heparin tubes for the measurement of hemoglobin (HGB) and hematocrit (HCT) using a KX-21 Automated Hematology Analyzer (Sysmex Corporation, Kobe, Japan). The remaining blood was centrifuged for 10 min at 3000 rpm in order to isolate plasma.

### Mitochondrial isolation

Mitochondrial isolation was performed as previously described [32, 33]. One aliquot of the crude mitochondrial fraction was used for protein determination. The remaining samples were then centrifuged at 13000 x g for 3 min at 4 °C. The mitochondrial pellets were suspended in an appropriate volume of medium C (1 M aminocaproic acid, 50 mM Bis-Tris-HCl [pH 7.0]) to obtain a protein concentration of 10 mg/ml.

### Plasma lipid peroxidation and mitochondrial organic HPx

The concentration of hydroperoxides (HPx), a specific and direct biomarker of lipid peroxidation, was determined using a Sigma PD1 kit (St Louis, MO, USA). Absorbance changes at 560 nm were monitored by spectrophotometry.

Blood collected from the bleeding procedure was centrifuged for 10 min at 3000 rpm to isolate the plasma. Then, 40 μl of plasma was used for the quantification of HPx concentration in plasma. A total of 100 μg of protein from the mitochondrial fraction was used to determine the mitochondrial concentration of organic HPx.

### Statistical analysis

Results are shown as the mean ± SD. Homoscedasticity and normality were tested by Levene’s test and the Kolmogorov-Smirnov test, respectively. Two-way repeated measures ANOVAs were used to analyze performance during the maximal velocity tests, as well as to analyze weekly and mesocycle work. The different groups represented the between-subjects variable, and time was the inter-subjects variable. A post hoc analysis was performed, and confidence intervals were adjusted using the Bonferroni correction when the effect was significant. One-way ANOVAs were used to analyze the remaining data. The level of significance was set at *p* < 0.05. All analyses were performed using the Statistical Package for Social Sciences (SPSS, version 22 for Windows; IBM Corp., Armonk, NY).

## Results

**Fig. 1:**
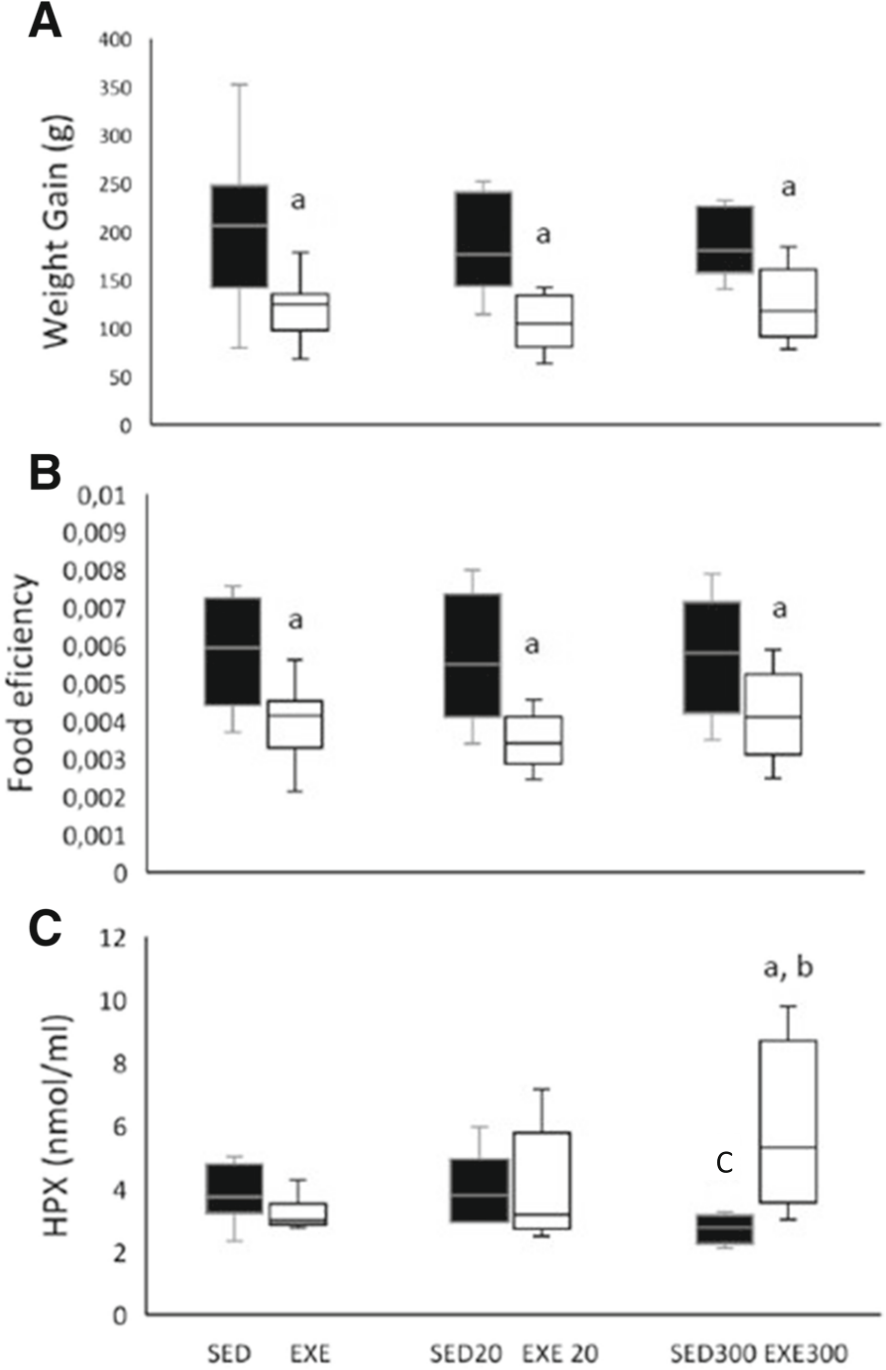
High hydroxytyrosol intake becomes pro-oxidant in exercised rats. Weight gain (**A**) and feed efficiency (**B**) reported throughout the study. Hydroperoxides (**C**) were quantified in plasma at the end of the study. ^**a**^*p* < 0.05 compared with the sedentary group. ^**b**^*p* < 0.05 compared with the EXE group. ^**c**^*p* < 0.05 compared with the SED group. SED, sedentary; EXE, exercised; 20, group supplemented with 20 mg/kg/d of hydroxytyrosol; 300, group supplemented with 300 mg/kg/d of hydroxytyrosol

**Fig. 2:**
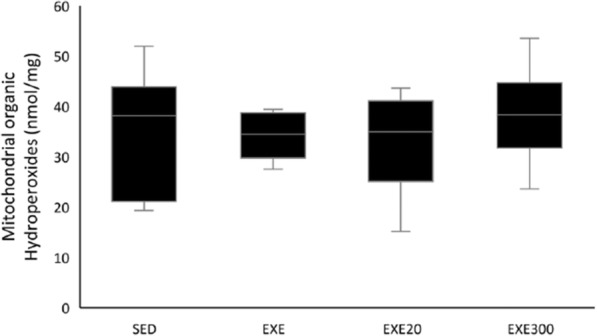
Mitochondrial organic Hydroperoxides. No statistical differences were found for mitochondrial organic hydroperoxides, SED, sedentary; EXE exercised; EXE20, group supplemented with 20 mg/kg/d of hydroxytyrosol; EXE300, group supplemented with 300 mg/kg/d of hydroxytyrosol

The one-way ANOVAs showed that weight gain (Fig. 1A) and feed efficiency (Fig. 1B) were unaffected by HT either in sedentary or in exercised rats throughout the study. However, exercise induced less weight gain and reduced feed efficiency in all the exercised groups (*p* < 0.05 for all groups and for both variables). The results (Fig. 1C) show that 300 mg/kg/d HT functions as antioxidant in the SED300 rats since this dose decreases the concentration of plasma HPx compared with the SED group (*p* < 0.05). However, we found that 300 mg/kg/d HT functions as a pro-oxidant agent in exercised animals. Indeed, the one-way ANOVA showed that the EXE300 group exhibited a higher plasma HPx concentration than the EXE and SED300 groups (*p* < 0.05 for both). Moreover, there was a trend (*p* = 0.09) toward a higher plasma HPx concentration in the EXE300 group compared with the EXE20 group. We isolated mitochondria from the gastrocnemius muscle of exercised rats, in order to determine the mitochondrial oxidative status. Despite organic mitochondrial HPx being 9%, 11% and 15% higher for EXE300 than for SED, EXE and EXE20 respectively, these results were not significant (Fig. 2).

One-way ANOVA was also used to analyze hematological data (Table 1). We found that the SED20 (*p* < 0.001) and SED300 (*p* < 0.05) groups had lower HGB levels than the SED group. Similarly, the SED20 group (*p* < 0.01) had a lower HCT than the SED group. The fact that the EXE300 group showed a lower HCT and lower HGB values (*p* < 0.05 for both) than the EXE20 group is noteworthy.

**Table 1:** Hematological parameters at the end of the study

	HGB (g/dl)	HCT (%)
Sedentary		
SED	14.7 ± 0.68	42.5 ± 2.09
SED20	10.8 ± 1.54***	36.7 ± 2.21**
SED300	12.7 ± 1.75*	40.3 ± 2.08
Exercised		
EXE	14.9 ± 0.88	42.0 ± 2.57
EXE20	15.2 ± 0.51	43.9 ± 2.36
EXE300	13.9 ± 0.37#	40.3 ± 2.02#

*HGB* hemoglobin, *HCT* hematocrit, *SED* sedentary, *EXE* exercised, *20* 20 mg/kg/d of hydroxytyrosol, *300,*300 mg/kg/d of hydroxytyrosol. * *p* < 0.05; ** *p* < 0.01; *** *p* < 0.001 compared to the SED group. # *p* < 0.05 compared to the EXE20 group

**Fig. 3:**
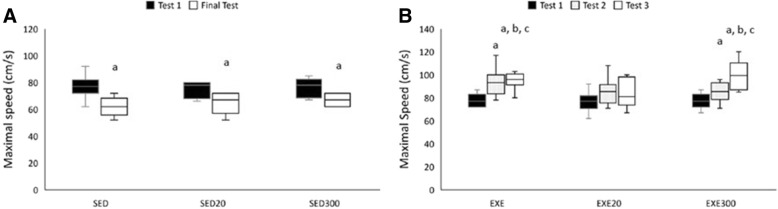
Maximal velocity performance is influenced by hydroxytyrosol. Maximal velocity tests in sedentary rats (**A**) were performed prior to and at the end of the study. The maximal velocity test in exercised animals (**B**) was performed before the study, after 5 weeks and at the end of the study. ^**a**^*p* < 0.05 compared to Test 1. ^**b**^*p* < 0.05 compared to Test 2. ^**c**^*p* < 0.05 compared to the SED20 group in Test 3. SED, sedentary; EXE, exercised; 20, group supplemented with 20 mg/kg/d of hydroxytyrosol; 300, group supplemented with 300 mg/kg/d of hydroxytyrosol

Two-way repeated measures ANOVA showed a significant group × time interaction (*p* < 0.001, eta^2^ = 0.645, 1- β = 0.999) when analyzing maximal running velocity (Fig. 3). A more thorough analysis showed that all sedentary groups lost their performance when comparing Test 1 with the final test (Fig. 3A; *p* < 0.05 for all groups). The analysis of the exercised animals is shown in Fig. 3B. The EXE and EXE300 groups increased their maximal velocity from Test 1 to Test 2 (*p* < 0.05) and a further increase was observed from Test 2 to Test 3 (*p* < 0.05). Both EXE (*p* < 0.05) and EXE300 (*p* < 0.05) groups showed a higher maximal velocity than the EXE20 group at the end of the study. Indeed, the maximal velocity of the EXE20 group remained unchanged during the three tests performed.

**Fig. 4:**
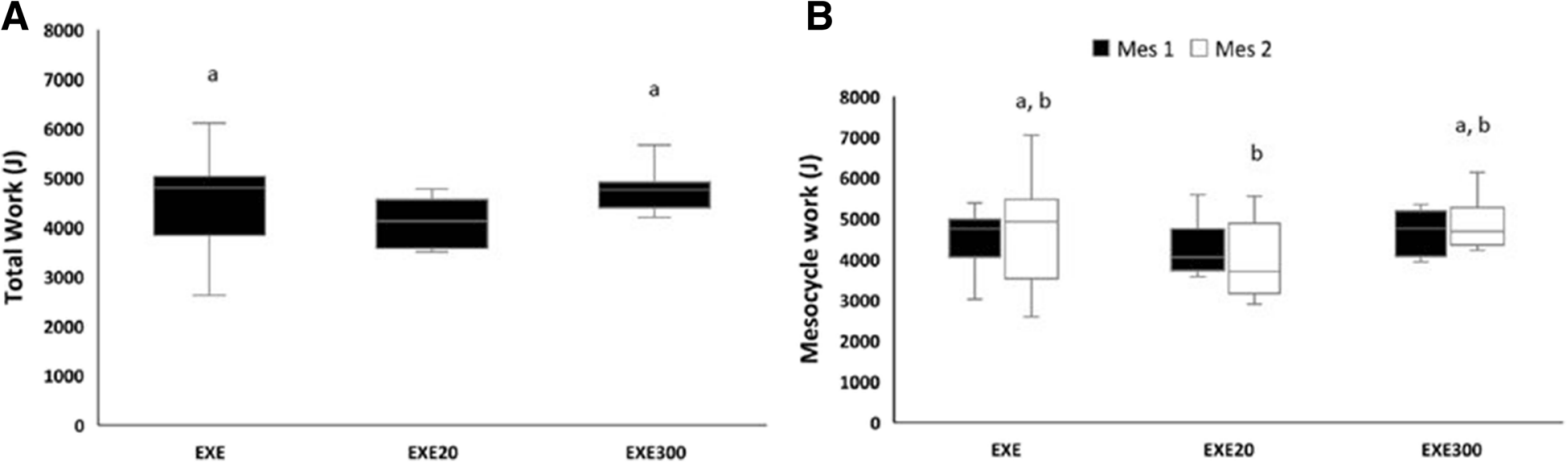
Hydroxytyrosol affects physical work performed during the training protocol. Total work (**A**) and mesocycle work (**B**). ^**a**^*p* < 0.05 compared to the EXE20 group. ^**b**^*p* < 0.05 compared to MES1. EXE, exercised; EXE20, EXE supplemented with20 mg/kg/d of hydroxytyrosol; EXE300, EXE supplemented with300 mg/kg/d of hydroxytyrosol; MES, mesocycle

Daily physical work was monitored during each training session (Fig. 4). One-way ANOVA showed that rats in the EXE20 group ran less than those in the EXE group (*p* < 0.001) and the EXE300 group (*p* < 0.001) when analyzing daily physical work throughout the entire protocol (Fig. 4A). Moreover, a significant group × time interaction (*p* = 0.038, eta^2^ = 0.155, 1- β = 0.622) was found when analyzing work performed during each mesocycle (Fig. 4B). Both the EXE and EXE300 rats increased (*p* < 0.05) their physical work from mesocycle 1 to mesocycle 2, while the EXE20 rats decreased their physical work (*p* < 0.05). Indeed, the EXE20 group performed less work (*p* < 0.05) than the EXE and EXE300 groups at mesocycle 2.

## Discussion

We aimed to describe the effects of a low and a high HT dose on the physical capacity and redox state of both sedentary and exercised rats. Our results suggest that 20 mg/kg/d of HT intake over 10 weeks may hinder training-induced physical capacity enhancement, whereas a dosage of 300 mg/kg/d HT does not. This effect may be related to changes in the systemic redox environment, as the 300 mg/kg/d dosage increases plasma HPx levels. All the supplemented groups (i.e., exercised and sedentary) showed similar weight gain, suggesting no evidence for potential toxicity for the doses used [28]. However, the lower levels of HGB together with the higher HPx levels reported in the EXE300 group should be further studied as they may reflect a harmful effect of the higher HT dose when combined with exercise.

The antioxidant capacity of the endurance exercise is well recognized. This systemic effect is achieved because within contracting muscles, there are multiple sites of ROS production, including the mitochondrial respiratory chain [34] and the plasma membrane [35]. These ROS act as signaling molecules that activate molecular pathways, which chronically lead to improvements in the function and content of endogenous antioxidants [15, 36]. It should be highlighted that the exercise protocol applied induces a strong antioxidant effect within skeletal muscles, which is reflected in blood plasma as a decrease in HPx concentration [29]. In this scenario, the loss of performance reported in the EXE20 group may be a consequence of the antioxidant effect of this dose. However, we have not found statistical evidence supporting that the 20 mg/kg/d dosage of HT has antioxidant effects. This occurred even though this dose was previously described as an antioxidant in rodents [21]. A plausible explanation is that the antioxidant effect induced by endurance exercise may mask any additional antioxidant effects induced by the low HT dose. Moreover, Feng et al. [20] showed that 25 mg/kg/d HT blunts the autophagic response of exercised rats. Importantly, the autophagic response to exercise is known to be ROS-dependent [37, 38]. Taken together, our results and those previously published suggest that dosages close to 20 mg/kg/d may hinder some exercise adaptations, probably due to their antioxidant effect.

An important finding of the present study is that 300 mg/kg/d of HT decreased plasma HPx concentrations in sedentary rats while it increased plasma HPx concentrations in exercised rats. It is unclear why this redox change toward a pro-oxidant effect occurs. However, a similar effect has been described for other polyphenols in vitro. As a by-product of their antioxidant activity, polyphenols can be oxidized [22, 24], and if the glutathione concentration is not high enough, the potential oxidative reactions may be focused on protein thiols [23]. Therefore, during the antioxidant activity of polyphenols such as HT, several metabolites are produced, and some of them may turn to pro-oxidant agents. This effect could be more easily manifested in the exercised rats consuming a high HT dose because exercise adaptations result are preceded by transient increases in oxidative stress. Indeed, it has been recently reported that subjects who show a higher oxidative response to a given training have a greater adaptation than those who show a lower level of oxidative stress [19]. Under this scenario, the polyphenol paradox may have increased the oxidative bursts for each training session and may explain the higher performance exhibited by the EXE300 animals.

It should be highlighted that we have not found evidence for mitochondrial redox alterations within exercised animals. Several potential explanations for the differences found between the plasma and mitochondrial compartments, especially for the EXE300 group, are suggested. First, mitochondria can alter their inner membrane structure in response to exercise in order to prevent their oxidative damage. Recent findings from our group showed that physical exercise stimulates the assembly of mitochondrial complexes in Supercomplexes (SCs), and prevents excessive ROS production [29]. Exercise can increase mitochondria resilience to excessive oxidative stress. In addition, previous studies on contracting muscles have reported that the main source of ROS production in response to contractile activity is the enzyme NADPH-oxidase, wich is mainly located in the sarcoplasmic reticulum [35]. Finally, data on other polyphenols suggest that plasma proteins and lipids can be rapidly oxidized in response to high polyphenol concentrations [39, 40]. Altogether, these data suggest that exercise training may adapt mitochondria to be protected from excessive oxidative stress. However, the high polyphenol dose in conjunction with the ROS produced by the extra mitochondrial enzymes may result in increases in circulating lipid peroxide.

Table 1 shows that sedentary animals consuming HT may undergo hematological dysfunction. However, Wistar rats are known to show high variability in response along hematological parameters. Previous studies on sedentary rats subjected to an intake of 25 mg/kg/d of the polyphenol quercetin showed lower hematological values than exercised rats, wich is consistent with the data of the present study. Indeed, HCT values below 35% and HGB values below 11 g/dL have been reported [41]. These results are consistent with the concept that polyphenols may interfere with circulating proteins [39, 40]. However, it seems that exercise can prevent such an effect but only when a low dose of HT is administered. In fact, we found a lower HGB concentration in the EXE300 group compared to the EXE20 group. Therefore, even though the EXE300 rats may have maintained their physical capacity, if HPx and HGB are considered in conjunction, these data may reflect the beginning of a harmful process. The optimal HT dose and the supplementation period required to maximize benefits (i.e., endurance performance) without inducing harmful effects must be elucidated in future studies. Furthermore, the high variability found in HGB and HCT data reported here and previously in response to polyphenol intake [41] suggests that Wistar rats may not respond homogenously to HT intake.

## Conclusions

In summary, HT dosages ranging from 20 mg/kg/d to 300 mg/kg/d for 10 weeks induced an antioxidant response in a dose-dependent manner in sedentary animals. However, 20 mg/kg/d HT decreased the running capacity when this dose was supplemented during exercise, whereas 300 mg/kg/d HT was able to maintain and even increase the running capacity. This effect might be due to a systemic pro-oxidant effect induced when a high HT dose is supplemented during exercise training. However, mechanistic studies should address the optimal HT doses and supplement duration necessary to increase physical capacity through inducing oxidative stress without resulting in any harmful effects.

## Abbreviations

EXE: exercised
HCT: hematocrit
HGB: hemoglobin
HPx: hydroperoxide
HT: hydroxytyrosol
ROS: reactive oxygen species
SED: sedentary
